# Evaluation of an electronic clinical decision support algorithm to improve primary care management of acute febrile illness in rural Cambodia: protocol for a cluster-randomised trial

**DOI:** 10.1136/bmjopen-2024-089616

**Published:** 2024-10-18

**Authors:** Rusheng Chew, Elke Wynberg, Marco Liverani, Huy Rekol, Chea Nguon, Lek Dysoley, Moul Vanna, James John Callery, Abhijit Mishra, Bipin Adhikari, Rupam Tripura, Arjun Chandna, Greg Fegan, Naomi Waithira, Richard James Maude, Nicholas P J Day, Thomas Julian Peto, Yoel Lubell

**Affiliations:** 1 Mahidol Oxford Tropical Medicine Research Unit, Bangkok, Thailand; 2 Centre for Tropical Medicine and Global Health, University of Oxford, Oxford, UK; 3 Faculty of Medicine, University of Queensland, Brisbane, Queensland, Australia; 4 London School of Hygiene & Tropical Medicine, London, UK; 5 National Center for Parasitology, Entomology and Malaria Control, Phnom Penh, Cambodia; 6 Action for Health Development, Battambang, Cambodia; 7 Cambodia Oxford Medical Research Unit, Siem Reap, Cambodia

**Keywords:** Clinical Trial, eHealth, Public health, Tropical medicine, Randomized Controlled Trial

## Abstract

**Introduction:**

Acute febrile illness (AFI), traditionally attributed to malaria, is a common reason for seeking primary healthcare in rural South and Southeast Asia. However, malaria transmission has declined while health workers are often poorly equipped to manage non-malarial AFIs. This results in indiscriminate antibiotic prescribing and care escalation, which promotes antibiotic resistance and may increase healthcare costs. To address this problem, an electronic clinical decision support algorithm (eCDSA) called ‘Electronic clinical Decision support for Acute fever Management (EDAM)’ has been developed for primary health workers which integrates clinical, epidemiological and vital sign data with simple point-of-care tests to produce a diagnosis and management plan.

**Methods and analysis:**

This is a pragmatic cluster-randomised trial aiming to assess the effect of EDAM and related training on antibiotic prescribing rates in rural Cambodian primary health centres (PHCs) as the primary outcome, along with a range of secondary outcomes including safety. Patients with AFI are eligible for recruitment if they are aged ≥1 year. A cluster is defined as a PHC and PHCs will be randomised to control (standard of care) and intervention (EDAM and associated training) arms, with 15 PHCs per arm. Patients will be followed up after 7 days to ascertain the safety profile of EDAM. Each PHC will recruit 152 patients (total 4560), based on a baseline antibiotic prescription rate of 25% and expected reduction to 17.5% with EDAM.

**Ethics and dissemination:**

Results will be published in international peer-reviewed journals to inform the design of future versions of EDAM and of future trials of similar eCDSAs and other digital health interventions targeted towards rural populations. This study was approved by the Oxford University Tropical Research Ethics Committee (550-23) and the Cambodian National Ethics Committee for Health Research (395-NECHR).

**Trial registration number:**

International Standard Randomized Controlled Trial Number Registry (ISRCTN15157105).

**STRENGTHS AND LIMITATIONS OF THIS STUDY**

- The clinical management plans which are part of the intervention were devised by Cambodian specialist doctors and reviewed and endorsed by the local health authority, thus ensuring their contextual appropriateness.
- The use of tablet devices for data collection ensures data quality since range checks, data field restrictions and skip logic will prevent missing data as well extreme values being entered.
- Intercluster and intracluster heterogeneity, as well as the diversity of rural primary care providers in low-income and middle-income countries, may affect the generalisability of the findings, since the trial is only being conducted in Cambodia among primary health centres with relatively high antibiotic prescribing rates.
- The primary outcome of reduction in antibiotic prescribing rates was chosen as it is objective and easily measurable, but assessment of the other benefits of the intervention, both tangible and intangible, is not as easily measured by the methodology used in this study.
- While the estimated effect size was based on the best available evidence, its accuracy is uncertain which, in turn, may affect the sample size calculations.

## Introduction

 Acute febrile illness (AFI) is a common reason for patient presentations to primary healthcare providers, such as primary healthcare centres (PHCs), in rural South and Southeast Asia.^1^ Malaria was previously a common cause for acute fever. However, the drastic reduction in malaria incidence in the region and the established use of malaria rapid diagnostic tests mean that presumptive treatment for malaria in such patients is no longer appropriate. Compounding this issue is poor clinical and laboratory diagnostic capacity due to scarcity of highly skilled health workers and access barriers to healthcare facilities. These factors often lead to suboptimal clinical decision-making, resulting in issues such as overprescription of empirical antibiotic therapy and missed identification of patients needing higher level care. In addition to the impact on the quality of patient care, overprescription of antibiotics also drives antimicrobial resistance (AMR), a problem which is especially prevalent in this region. The World Health Organization (WHO) has previously identified the threat of AMR to human health in the Southeast Asian region to be one of the highest in the world,^2^ with the Global Research on Antimicrobial Resistance project estimating that approximately 389 000 and 254 000 deaths in the South Asia and Southeast Asia regions, respectively, were directly attributable to AMR in 2019.^3^

Previous work in South and Southeast Asian low-income and middle-income countries (LMICs), and LMICs elsewhere, has assessed the efficacy and cost-effectiveness of simple tools to aid clinical decision-making for AFI, such as pulse oximetry and C reactive protein (CRP) rapid tests.^4^,^7^ The use of algorithms based on clinical symptoms and signs, such as the WHO Integrated Management of Childhood Illness, is also well established. However, these algorithms are only relevant to children <5 years old and have limited reach electronically. Electronic clinical decision support algorithms (eCDSAs) integrating point-of-care diagnostics, pulse oximetry and symptom-based algorithms accessed on mobile devices may be a more efficient and user-friendly way of deploying such clinical decision support assistance to rural PHCs, with encouraging results from one study in sub-Saharan Africa.^8^ However, to date, no such eCDSA tailored to rural South and Southeast Asian primary care settings has been developed or assessed under real-world conditions. This may be due to barriers noted in evaluations of eCDSAs elsewhere, such as lack of technical skills among healthcare workers, heavy workloads, perceived threats to healthcare worker autonomy, and inability of the eCDSA to be integrated into clinical workflows effectively.^9 10^ Lack of patient trust in eCDSA-generated recommendations and concerns regarding data privacy and security may also be additional inhibiting factors.^11^

The South and Southeast Asian Community-based Trials Network (SEACTN), headquartered at the Mahidol Oxford Tropical Medicine Research Unit (MORU) in Bangkok, Thailand has been in operation since 2021, and in its first years has aimed to define the regional epidemiology of AFI in rural populations across the region.^12^ As of October 2023, over 83 000 patients of all ages presenting with AFI in primary care settings have been recruited across five countries (Thailand, Bangladesh, Laos, Cambodia and Myanmar). Patient enrolment was performed by health workers in PHCs and by village health workers. As part of this study, detailed data on clinical symptoms and vital signs were collected, allowing formulation of age-appropriate syndromic diagnoses using algorithms developed by a committee of experts comprising an adult infectious diseases and respiratory physician (RC), an adult general and infectious diseases physician (NPJD), an adult general physician (AC), a paediatric infectious disease specialist (Prof Enitan Carrol, University of Liverpool, UK), and a general practitioner (JC), all of whom have extensive experience working in rural low-income and middle-income country (LMIC) settings.

These syndromic decision rules have been integrated with malaria and CRP rapid tests, pulse oximetry and management plans derived from local specialist clinical expertise and/or local guidelines (where these exist) to create an eCDSA called (Electronic clinical Decision support for Acute fever Management (EDAM) for the diagnosis and management of acute fever in South and Southeast Asia. EDAM takes the form of an app which can be deployed on mobile devices with the capability to function without an Internet connection. This effort has been motivated by the appetite among experts, policy-makers and healthcare workers in PHCs for algorithmic management of AFI incorporating point-of-care tests, as documented in a series of stakeholder analyses conducted across SEACTN countries, for example, in Laos by Liverani *et al*,^13^ with other manuscripts in preparation, and as described in the literature.^14 15^ Of note, CRP rapid tests and pulse oximetry are currently not routinely used as part of the primary care management of AFI in the settings where this trial is to be run. Besides being the first eCDSA for AFI in the rural Southeast Asian primary care context, and by incorporating point-of-care diagnostic tools other than malaria rapid tests and having antimicrobial stewardship as a guiding principle in its development, EDAM is an improvement on current guideline-based eCDSAs for AFI and is in line with the target product profile for such eCDSAs proposed by the Foundation for Innovative New Diagnostics.^16^

This cluster-randomised trial aims to determine whether EDAM can improve routine clinical management of patients with AFI, primarily in terms of more appropriate antibiotic prescribing. As part of the study, the usability and acceptability of EDAM for healthcare workers and the cost-effectiveness of EDAM compared with routine care will also be assessed, but these components do not form part of this study protocol which focuses solely on the conduct of the trial. The results will provide a solid foundation for future iterations of the app, which may include more sophisticated technologies such as machine learning as has been done for eCDSAs for AFI in Nigeria.^17^,^19^

## Methods and analysis

### Design

This is a pragmatic, cluster-randomised controlled trial with two study arms consisting of 15 PHCs in the intervention (EDAM-guided clinical management, further details of which are provided below) arm and 15 PHCs in the control (standard care) arm. A PHC will be considered as a cluster, and the assignment of PHCs to arms will be randomly performed by computer. The pragmatism of the study was confirmed using the PRagmatic Explanatory Continuum Indicator Summary−2 tool,^20^ with the majority of the study design domains rated ‘very pragmatic’ (online supplemental information S1).

The rationale for this study design is that any study assessing the potential performance of eCDSAs like EDAM must necessarily be pragmatic and conducted in environments as similar to real-world conditions as possible. Otherwise, in a research-oriented context, healthcare worker prescribing and other management practices may be consciously or unconsciously affected. Furthermore, for policy-makers to consider wide-scale eCDSA implementation, what is most needed is a pragmatic implementation study of its impact in routine care. As such, this study will evaluate the introduction of EDAM into routine care with no research staff routinely present. To minimise disruption and alteration of routine care, a waiver of written patient informed consent was requested from the relevant ethics committees, in accordance with the 2016 WHO/CIOMS International Guidance for Health-related Research Involving Humans. In brief, the 2016 CIOMS Guideline 10 states that in the following circumstances a waiver of informed consent could be applied^21^:

- if the research would not be feasible or practicable to carry out without the waiver or modification;
- if the research has important social value; and
- if the research poses no more than minimal risks to participants.

The proposed study and intervention meet all of these criteria. The infeasibility of obtaining extensive informed consent from each potential participant by research staff has already been explained. In support of this argument, Sim and Dawson, and Dal-Ré and colleagues, state that waiving informed consent is justifiable when the methodological integrity of the study is brought into question.^22 23^ In order for healthcare systems to be responsive and improve patient care implementation, studies are required to generate evidence for policy change and to ensure that the benefits seen in research settings are translated into routine care. There is precedent for this waiver, as illustrated by a similar cluster-randomised trial performed recently in Vietnam.^4^ Thus, instead of obtaining written informed consent, which would also disrupt patient care, verbal assent will be obtained prior to the performance of rapid malaria diagnostic and CRP tests, as is usual in clinical practice, and to allow follow-up by research staff. The drawing of fingerprick blood for malaria and CRP rapid tests is also of minimal risk and the blood taken is only being used for direct patient care. Similar to other tests that may be indicated, patients or their legal guardians will be able to refuse these tests, without any other impact on their care.

The research question is of high clinical importance and social value, helping to guide more appropriate antibiotic prescribing by both reducing unnecessary antibiotic prescribing in the community, a key driver of AMR,^24 25^ and identifying patients who need referral to hospital and/or antibiotics but may be missed by routine clinical assessment. The EDAM app offers these potential improvements with a low-risk intervention, that could be rapidly scaled up. Indeed, the components of EDAM (which are described fully below and include pulse oximetry, malaria and CRP rapid tests combined with clinical symptom-based guidelines) have each individually been determined to be of minimal risk and are already widely used for these purposes in other settings. Furthermore, large clinical trials of similar, but not identical, eCDSAs with extensive patient follow-up have been shown to be effective in primary care settings elsewhere.^8 26^

### Setting

The study will be conducted in Cambodia, where PHCs deliver most of the primary care services and national targeted health programmes to the population, especially in rural areas. Apart from acute care, services provided by PHCs include hygiene, vaccinations, antenatal care, safe child delivery and health education.

The PHCs will be selected from among those in rural areas of Battambang province, through another longstanding collaboration with the non-governmental organization (NGO) Action for Health Development (AHEAD).

PHCs selected to participate in this study must meet all the following conditions:

- Have healthcare workers authorised to prescribe antibiotics.
- Stock antibiotics.
- Are likely to have at least 150 patients present with AFI within a 4-month to 6-month period, verified by checking patient logbooks and/or numbers recruited into the SEACTN observational study.

### Participant characteristics

Healthcare workers will be advised that use of EDAM should be restricted to patients within a recommended target population. This target population comprises patients meeting all the following inclusion and exclusion criteria.

The inclusion criteria are as follows:

- Age ≥1 year.
- Unscheduled presentation for acute care.
- Documented fever (≥37.5 °C axillary) or hypothermia (<35.5 °C) or history of fever in the last 24 hours.

The exclusion criteria are as follows:

- Onset of illness >14 days.
- Presenting due to accident or trauma.
- Presenting ≤3 days after routine immunisations.
- Presenting within the follow-up period.

Should a patient not meet all criteria, enrolment will not be permitted by the internal logic of the EDAM app. The app will advise healthcare workers in intervention arm PHCs to continue standard practice for non-eligible patients.

Investigators may discontinue a participant from the study at any time if considered necessary for any reason, including but not limited to:

- Ineligibility (either arising during the study or having been overlooked during screening).
- Significant protocol deviation.
- Significant non-compliance with study requirements.

The reason for withdrawal will be recorded. Data from withdrawn participants will be included in the intention-to-treat (primary) analysis but not in any per-protocol analyses.

Participants have the right to withdraw from follow-up at any time.

### Description of intervention

The intervention is the EDAM app deployed on tablet devices, which uses data on clinical features including vital signs and pulse oximetry with malaria and CRP rapid tests to guide primary healthcare workers in their clinical decision-making when confronted with patients with AFI, plus training required for effective use of the app. This training will include familiarisation with the app interface, as well as measurement and interpretation of vital signs, CRP rapid tests and pulse oximetry.

For the purposes of this study, the EDAM app was constructed using the CommCare platform (Dimagi, Cambridge, USA). This platform was chosen for familiarity reasons among primary healthcare workers with an established link to SEACTN, as it was also used to construct the electronic data collection tool used in SEACTN.

As part of screening, patients presenting to a PHC in the intervention arm will have their demographic details logged on the EDAM app. Assessment of study eligibility will be performed by a healthcare worker. If the patient is eligible for enrolment, vital signs, including measurement of peripheral oxygen saturation using pulse oximetry, will be performed. One of three possibilities may then ensue:

1. The patient meets eligibility criteria and has no danger signs, either measured by the health worker or observed by any of the health worker, patient or caregiver (table 1). In this case, the patient will be enrolled and EDAM will guide the healthcare worker through the in-built algorithm leading to a suggested initial management plan, which will include a malaria rapid test as per local policy. The selection of relevant observed danger signs (e.g., convulsions) was based on established national and international clinical guidelines and ratified by a panel of local expert clinicians. All management plans were also devised by local expert clinicians with regard to local guidelines and approved by the relevant health authority.
2. The patient meets eligibility criteria and has one or more measured or observed danger signs. In this case, EDAM will recommend a malaria rapid test and empirical antibiotic prescription (the choice of which will depend on local guidelines) and immediate referral to hospital. The patient will be enrolled and the algorithm will end here.
3. The patient does not meet eligibility criteria. In this case, EDAM will inform the healthcare worker that it is not appropriate to be used for this patient and that they should revert to their usual practice in dealing with non-acutely febrile patients. The patient will not be enrolled and the algorithm will end here.

**Table 1:** Measured and observed danger signs by age group for patients enrolled into the study

Age group	Measured danger signs	Observed danger signs
Children aged ≥1 to <5 years	Voice/pain/unresponsive on Alert, Verbal, Pain, Unresponsive (AVPU) scale SpO_2_<90% Severely abnormal heart rate (pulse of <80 or >160 beats per minute)	Unable to drink anything or breastfeed Vomiting everything Generalised convulsions Unable to sit upright/stand/walk (when previously able)
Children aged 5 to <15 years	Voice/pain/unresponsive on AVPU scale Severely abnormal heart rate (pulse of <70 or >150 beats per minute (5 to <12 years) or <50 or >130 beats per minute (12 to <15 years)) Fast breathing (respiratory rate of ≥40 breaths per minute (5 to <12 years) or ≥30 breaths per minute (12 to <15 years)) SpO_2_<90%	Unable to drink anything Vomiting everything Generalised convulsions Unable to sit upright/stand/walk independently New-onset confusion
Adults aged ≥15 years	Voice/pain/unresponsive on AVPU scale Severely abnormal heart rate (pulse of <40 or >140 beats per minute) Fast breathing (respiratory rate of ≥35 breaths per minute) SpO_2_<90% Systolic blood pressure <90 mm Hg	Unable to drink anything Vomiting everything Generalised convulsions Unable to sit upright/stand/walk independently New-onset confusion

SpO_2_, peripheral oxygen saturation

Intervention arm PHCs will each be provided with two sphygmomanometers and two pulse oximeters to mitigate the possibility of equipment failure. Nevertheless, where the algorithm requires a measurement by a machine, it will include an option not to enter a result.

Eligible patients will have a malaria rapid test administered early following measurement of vital signs, as is routine for all febrile patients at the study sites. This is based on the recommendation of the Cambodian National Malaria Control Program, since a continued comprehensive testing strategy is crucial to eradication efforts. If the test is positive, they will be treated with an antimalarial, the choice of which is dependent on local context. The structured approach of EDAM, however, as well as its capability to formulate diagnoses and recommend management for non-malarial AFI, extends the current paradigm of malaria-focused AFI management.

CRP rapid tests will be recommended to guide antibiotic prescription at appropriate points in the eCDSA in patients with no danger signs. The tests used in the trial are semiquantitative with threshold values of 10, 40 and 80 mg/L. If a CRP test is recommended and performed, the following guidance will be given to the healthcare worker:

- CRP <10 mg/L: reassure the patient that antibiotic prescription is not indicated.
- CRP >80 mg/L: prescribe antibiotics, the choice of which will depend on local contexts.
- CRP 10–80 mg/L: reassure the patient that antibiotic prescription is not indicated, unless there is concern based on the clinical judgement of the healthcare worker that the patient would suffer an adverse outcome if antibiotics were not prescribed.

**Figure 1:**
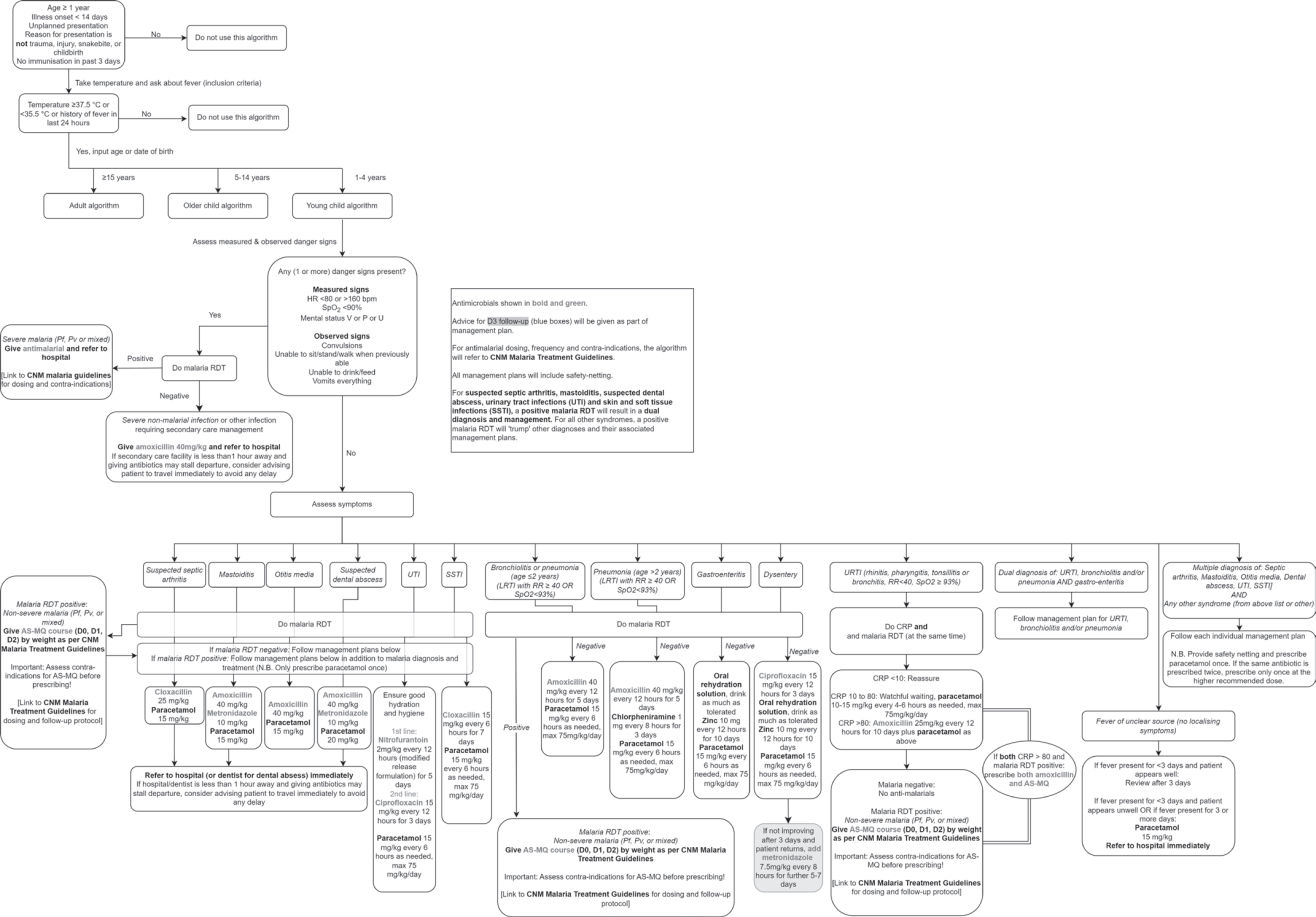
Schematic overview of the algorithm for children aged ≥1 year to <5 years. AS-MQ, artesunate-mefloquine; CNM, National Centre for Parasitology, Entomology and Malaria Control; CRP, C reactive protein; HR, heart rate; LRTI, lower respiratory tract infection; Pf, *Plasmodium falciparum*; PHC, primary health centre; Pv, *Plasmodium vivax*; RDT, rapid diagnostic test; RR, respiratory rate; SSTI, skin and soft-tissue infection; SpO_2_, peripheral oxygen saturation; URTI, upper respiratory tract infection; UTI, urinary tract infection; V or P or U, responds to voice or pain or is unresponsive.

Schematic overviews of the algorithms for children aged ≥1 to <5 years, children aged 5 to <15 years, and adults aged ≥15 years are shown in figures1^3^, respectively.

**Figure 2:**
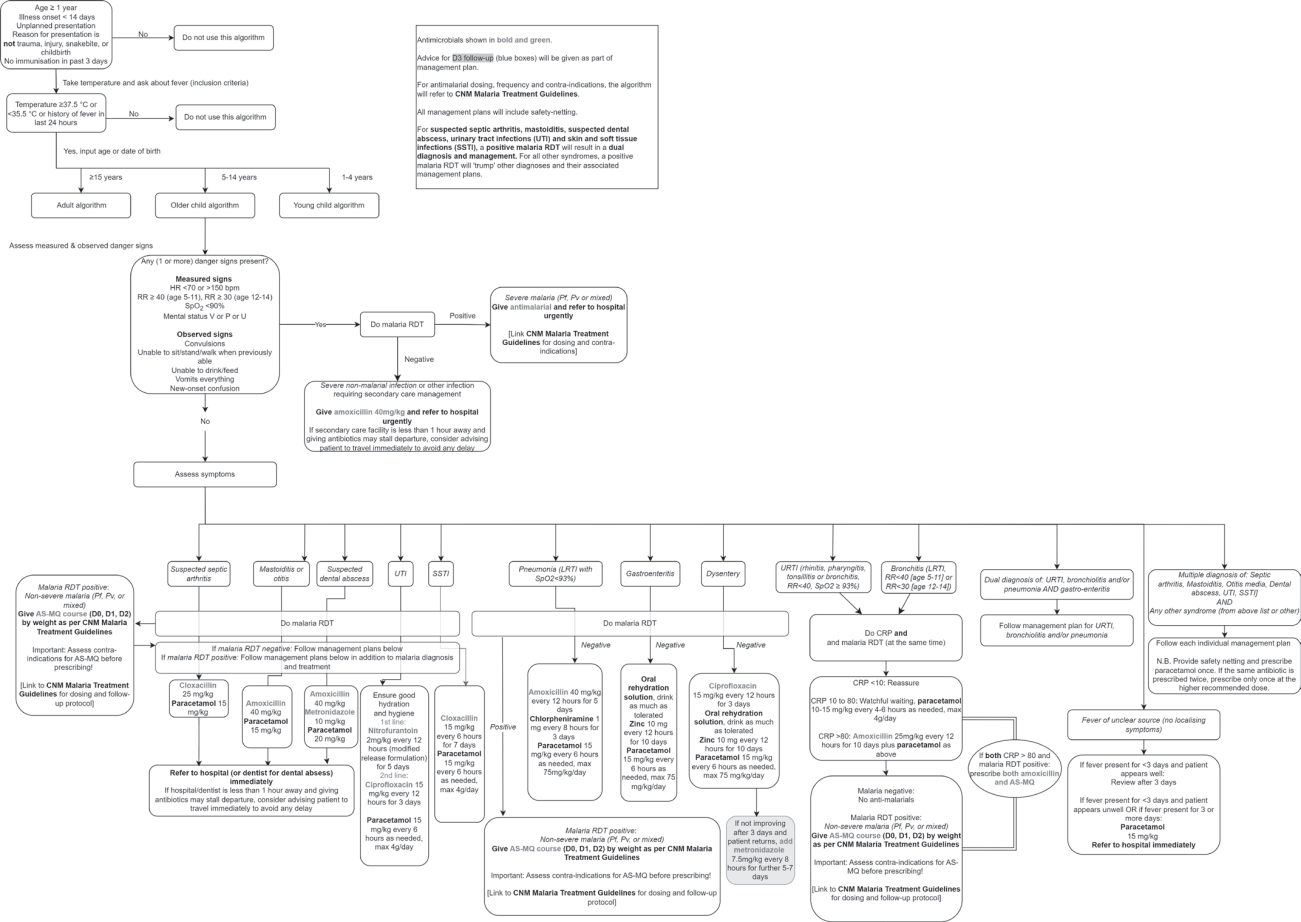
Schematic overview of the algorithm for children aged 5 years to <15 years. AS-MQ, artesunate-mefloquine; CNM, National Centre for Parasitology, Entomology and Malaria Control; CRP, C reactive protein; HR, heart rate; LRTI, lower respiratory tract infection; Pf, *Plasmodium falciparum*; PHC, primary health centre; Pv, *Plasmodium vivax*; RDT, rapid diagnostic test; RR, respiratory rate; SSTI, skin and soft-tissue infection; SpO_2_, peripheral oxygen saturation; URTI, upper respiratory tract infection; UTI, urinary tract infection; V or P or U, responds to voice or pain or is unresponsive.

**Figure 3:**
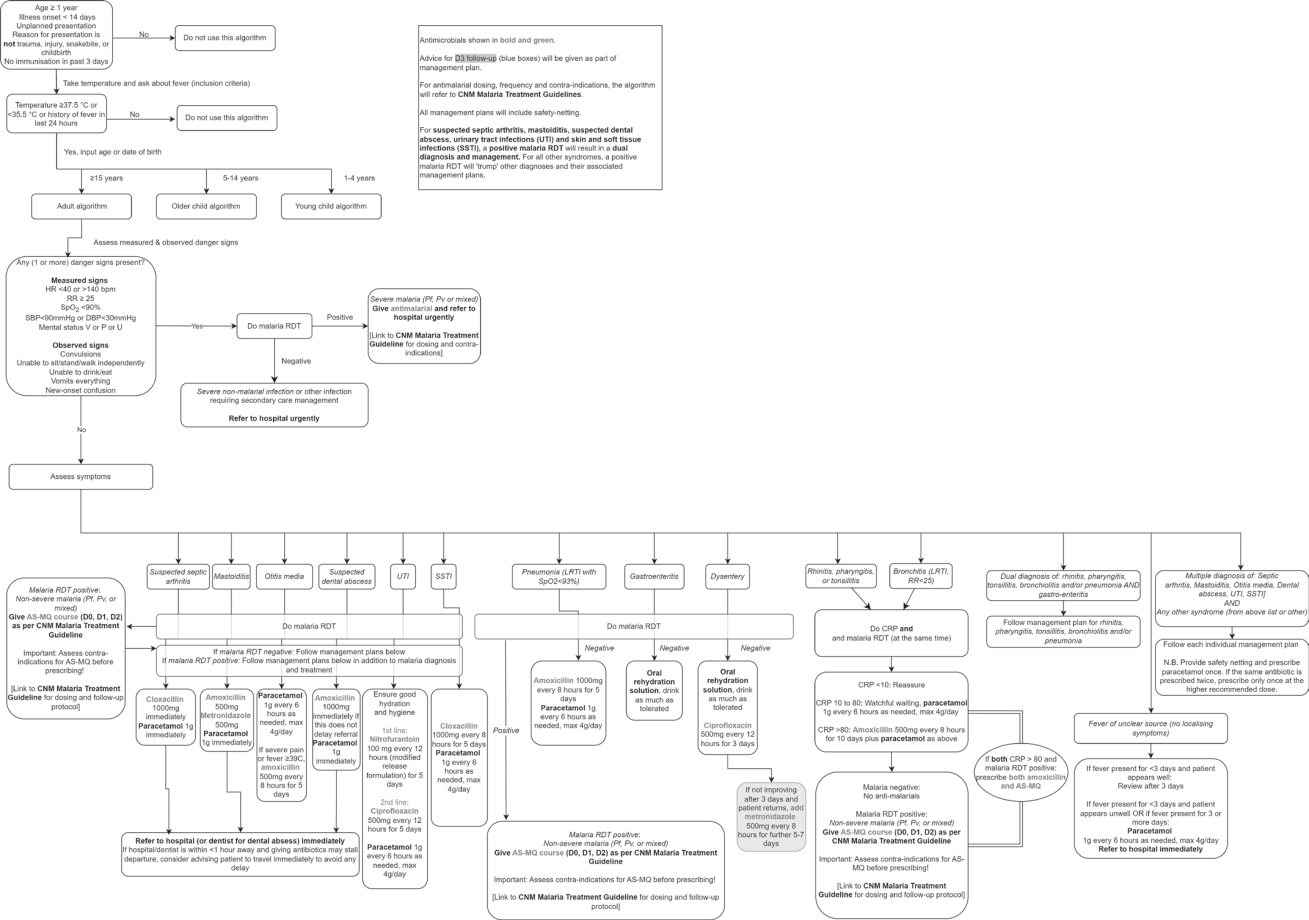
Schematic overview of the algorithm for adults aged ≥15 years. AS-MQ, artesunate-mefloquine; CNM, National Centre for Parasitology, Entomology and Malaria Control; CRP, C reactive protein; DBP, diastolic blood pressure; HR, heart rate; LRTI, lower respiratory tract infection; Pf, *Plasmodium falciparum*; PHC, primary health centre; Pv, *Plasmodium vivax*; RDT, rapid diagnostic test; RR, respiratory rate; SSTI, skin and soft-tissue infection; SBP, systolic blood pressure; SpO_2_, peripheral oxygen saturation; URTI, upper respiratory tract infection; UTI, urinary tract infection; V or P or U, responds to voice or pain or is unresponsive.

Safety netting will be recommended for all patients in the intervention arm not being referred, and healthcare workers in both arms will be trained on how to do this.

Healthcare worker autonomy in the intervention arm will be maintained, in that they will be allowed to use their discretion and clinical judgement regarding the suggested diagnoses and initial management. EDAM is not intended to be prescriptive, but merely a guide to help the majority of healthcare workers in their decision-making. This will be reflected in the wording of recommendations within the app. It is recognised that there may be healthcare workers who are more experienced or have higher skill levels than others, and that decision-making may be influenced by factors other the objective ones considered in EDAM. Where a healthcare worker does not agree with the suggested diagnosis and management, the app will collect data on their reasons so that these can be analysed with the goal of improving future versions of EDAM. It will be emphasised to healthcare workers that overriding the EDAM recommendation is entirely acceptable and that collecting information on the reasons for this will provide important insights for future improvement of the app and algorithm.

Healthcare workers in intervention arm PHCs will be provided with face-to-face training on the use of EDAM. They will also be taught how to use pulse oximeters and CRP rapid tests, which are not available in control arm PHCs, as well as trained on measuring blood pressure, respiratory rate and pulse rate, and assessing level of consciousness. Training will be conducted by the study team in the relevant local language prior to patient recruitment, with refresher training conducted on the advice of site coordinators or study monitors. Contact details of the study coordinator and/or field supervisor will be provided to PHCs; these personnel will be available for advice should any queries regarding the study arise.

To maintain clinical practice and workflows at control arm PHCs as close to the current standard of care as possible, the only extra activity for the trial required of healthcare workers in the control arm is screening and data collection. They will receive a simplified version of the EDAM app which will allow them to log patient demographic details as well as conduct patient screening and enrolment, but does not contain the clinical decision support algorithms present in the version used in the intervention arm. Control arm healthcare workers will receive training in the use of the simplified EDAM app, but not in the clinical procedures and use of point-of-care aids that healthcare workers in the intervention arm are taught.

### Description of other study procedures and follow-up

Only patients presenting acutely to study PHCs, that is, those without scheduled appointments, will be screened for eligibility. The app will use skip logic and contain prompts for the healthcare worker to check patient eligibility, allowing recruitment and data collection only when the requisite conditions are met.

Patients can be enrolled multiple times during the study period, as the primary objective is to determine the proportion of presentations which result in antibiotic prescription. However, they cannot be re-enrolled if they are within the follow-up period (defined below), as any attendances within this period will be logged as unscheduled re-presentations; this is a secondary outcome measure. If all the following identifiers of a patient are exact matches with those of another patient currently under follow-up, the healthcare worker will confirm that they are not the same person: date of birth (or age if date of birth is not known), gender and village of residence. Only once this confirmation is provided can the patient be enrolled.

All patients will have a medical history taken and will be examined as per normal practice. In intervention arm PHCs, this process will be more structured as it will be guided by EDAM. Practice will remain unchanged in control arm PHCs.

Patients will be followed up by telephone at 7 days post presentation (allow +2 days); patients who have not recovered by day 7 will be followed up at day 14 (+2 days). The following data will be collected at follow-up:

- Recovery (self-reported).
- If not recovered, whether symptoms were better, worse or the same.
- Whether they re-presented to another healthcare provider (re-presentations to the same PHC should be captured by the EDAM app).
- If they re-presented, whether they were hospitalised and (if known) whether antibiotics were prescribed.

Should a patient be uncontactable by telephone after three attempts, SEACTN village health workers will be used to locate the patient and conduct the follow-up interviews at day 7 and day 14, as necessary, if the participant survived, and document death, if the participant did not survive. If this is unsuccessful, the patient will be deemed lost to follow-up.

One important caveat is that patients who re-present during the follow-up period will be considered as unscheduled attendances secondary to the initial diagnosis, given that unscheduled attendance is a secondary outcome measure. This will also allow determination of whether antibiotics are initially withheld and then given at a subsequent visit. The major limitation of this approach, however, is that some patients may be categorised as unscheduled presentations when their subsequent visit is actually for a new problem or illness.

The trial schedule of enrolment, interventions and assessments, completed using the Standard Protocol Items: Recommendations for Interventional Trials (SPIRIT) guideline template,^27^ is shown in table 2. The completed SPIRIT checklist can be found in online supplemental information S2.

**Table 2:** Schedule of enrolment, interventions and assessments

Timepoint (DAY)	Study period			
	Enrolment	Clinical encounter	Follow-up	
	0	0	7	14
Enrolment:				
Eligibility screen	X			
Interventions:				
EDAM app		X		
Standard of care		X		
Assessments:				
Antibiotic prescription		X		
Hospital referral		X		
Recovery by day 7			X	
Recovery by day 14 (if not recovered by day 7)				X
Death or hospitalisation* by day 7			X	
Death or hospitalisation* by day 14 (if not recovered by day 7)				X
Re-presentation by day 7			X	
Re-presentation by day 14 (if not recovered by day 7)				X
Antibiotic consumption† by day 7			X	
Antibiotic consumption† by day 14 (if not recovered by day 7)				X

* If not referred to hospital at the time of clinical encounter.

† If not prescribed antibiotic at the time of clinical encounter.

EDAM, Electronic clinical Decision support for Acute fever Management

The trial commenced in May 2024. The end date is dependent on the rate of patient enrolment but will be no later than May 2025.

### Description of rapid tests to be used

Malaria rapid tests are already available at all PHCs as part of standard care. In addition, semiquantitative lateral flow CRP tests will be available for healthcare workers to use on eligible patients at intervention arm PHCs when prompted by EDAM. If a patient agrees to these tests, a finger-prick blood sample will be taken by the healthcare worker and placed onto the test kit in accordance with manufacturer instructions. Refusals will be recorded in the EDAM app. Other than these finger prick samples as part of the clinical management algorithm, no other samples will be taken. CRP test kits and associated consumables will be restocked on a monthly basis and checked to confirm they are properly used for study purposes only. The training for healthcare workers will emphasise respect for patient autonomy and that refusal on their part to be tested should be accepted without reservation. Reasons for patient refusal to be tested will be noted in the app to inform future algorithm development and community engagement activities.

The CRP rapid test which will be used is the Actim CRP test from Medix Biochemica (Finland; ISO certification ISO13485:2016).^28^ Much like a malaria rapid test, it is a simple lateral flow device that uses capillary blood, obtained through a finger-prick or heel-prick and provides a semiquantitative indication of whether CRP concentrations are <10 mg/L, between 10 and 40 mg/L, between 40 and 80 mg/L or >80 mg/L, in under 5 min with minimal training requirements. It meets European Union standards (CE-marking) as well as the regulatory requirements of 13 other countries and has been validated for accuracy in previous studies.^26 29^ The pulse oximeter which will be used is a Lifebox-Smile Train hand-held device, which is specifically manufactured to be durable for use in resource-limited settings,^30^ and which has been successfully used in SEACTN. Lifebox is a non-governmental organisation whose goal is to improve anaesthesia safety through the distribution of pulse oximeters developed for use in low-resource settings. Medix Biochemica and Lifebox played no role in the design of this study, and the investigators declare no conflicts of interest with respect to the choice of these point-of-care aids.

### Outcome measures

The primary outcome measure is the proportion of patients with AFI who are prescribed antibiotics in routine primary healthcare at initial presentation in the intervention and control arms.

Several secondary outcome measures will also be assessed:

1. The proportion of patients with full recovery at 7 and 14 days in intervention and control PHCs.
2. The proportion of patients referred to hospital at presentation in intervention and control PHCs.
3. The proportion of patients in intervention PHCs whose management followed the EDAM recommendations.
4. The proportion of patients with unplanned re-presentations to any healthcare facility at 7 and 14 days (if not fully recovered by 7 days) in intervention and control PHCs.
5. The proportion of patients prescribed antibiotics by a healthcare provider or who independently purchased antibiotics during the follow-up period, as determined by self-report, in intervention and control PHCs.
6. The proportion of patients with severe clinical outcomes (death or hospitalisation) at 7 and 14 days (if not fully recovered by 7 days) in intervention and control PHCs, not including those referred to hospital at presentation.
7. The proportion of patients prescribed an antibiotic in intervention PHCs by CRP level (<10 mg/L, between 10 and 80 mg/L, >80 mg/L).
8. The usability and acceptability of EDAM for healthcare workers (a separate ethical approval application will be made for this work).
9. The cost-effectiveness of EDAM compared with routine care.

Interpretation of the outcomes will consider the contextual factors inherent to the Cambodian primary care landscape, as described by Chew *et al*.^31^ In addition, the usability and acceptability of EDAM will be measured using structured interviews conducted with the healthcare workers in the intervention arm; this study does not form part of this protocol and will have its own ethical approval, as mentioned previously.

### Pre-trial and post-trial engagement

To gauge early policy-maker views on the potential usability and acceptability of the intervention, engagement meetings were held with key stakeholders in the Ministry of Health responsible for medical services, primary care, public health, and digital health and health informatics. Similar meetings were held with medical specialists in the field of adult internal medicine/infectious diseases, paediatrics and primary care to develop consensus clinical management plans tailored to local conditions as part of the algorithms. These management plans, as well as the overall design of the study, were further reviewed and endorsed by senior medical staff of the Battambang Provincial Health Department, and support for the trial was also obtained from the provincial government.

Taking into consideration evidence from similar eCDSAs used in other LMIC settings, such as ePOCT and inSCALE in sub-Saharan Africa,^8 32^ both groups were also asked for their views on the interface to inform the development of the EDAM app, which was then further refined by asking a small number of PHC healthcare workers to test the app and offer their opinions on its usability. These stakeholder consultations have informed the version of the EDAM app used in this study. A second round of such meetings will be conducted towards the end of the study to assess stakeholder experiences.

Before randomisation of participating PHCs, an overview of the study will be presented to all PHC healthcare workers. Education will also be provided about the role of antibiotics, AMR and good clinical practice (GCP) for research. Training specific to the study intervention has been described previously. Day-to-day field activities and monitoring will be undertaken by staff of MORU, AHEAD and the Provincial Health Department.

### Patient and public involvement

Information on EDAM for patients will be displayed in all study PHCs in the form of posters; however, patients were not involved in the design, conduct, reporting or dissemination plans of the study. Nonetheless, local engagement was undertaken to the extent possible via the activities described above.

### Sample size calculations

There is scant reliable routinely collected data on antibiotic prescription in the study PHCs. The baseline rate of antibiotic prescription was based on the findings from SEACTN, which includes PHCs where the proposed trial will be run,^12^ where data on patients prescribed antibiotics for AFI have been systematically collected since 2021. In the PHCs participating in this planned interventional study, the mean proportion of acutely febrile patients prescribed antibiotics was approximately 25%. The ICAT cluster-randomised trial, in which the proportion of patient recruited by age group was broadly similar to that seen in SEACTN, showed that the use of CRP rapid tests reduced the antibiotic prescription rate by 36%.^4^ Therefore, it is estimated that EDAM will result in a reduction of antibiotic prescription from 25% to 17.5%.

PHCs participating in SEACTN will also participate in this study if they meet criteria for inclusion. Other PHCs will be selected for inclusion in this study, if required, to make up a total of 15 PHCs in each of the control and intervention arms per country, on the assumption that their antibiotic prescription rates are also broadly similar. The PHCs will be drawn from across three Operational Districts as defined by the Battambang Provincial Health Department, with 10 selected from each operational district. As mentioned previously, each PHC will be treated as a cluster. Given the above, the sample size calculations are based on probabilities of 0.05 and 0.2 for type I and type II errors, respectively, and an intraclass correlation coefficient of 0.025, as is common for cluster randomised trials of this nature.^6 33^ Based on the parameters mentioned previously, with 15 clusters per arm the target number of patients to be recruited per cluster is 152 while assuming a drop-out rate of 10%. In total, therefore, the target number of patients to be recruited is 4560. Sample size calculations were performed in Stata V.18.

Stratified cluster randomisation of PHCs by operational district was performed; as mentioned previously, 10 clusters were included per district. A pseudorandomisation list of allocations to one of two possible arms (‘A’ or ‘B’) was generated using the *ralloc* package in Stata V.18.0 (StataCorp). This list was merged 1:1 with a list of PHCs. Assignment of arms ‘A’ and ‘B’ as either intervention or control was performed using the flip of a coin, with arm ‘A’ designated as the intervention arm if the coin flip came up heads and vice versa.

The study is not powered to detect differences within each age group, but subgroup analyses will be conducted among patients aged ≥1 to <5 years (younger children), 5 to <15 years (older children) and ≥15 years (adults). While inclusion of safety endpoints as coprimary objectives was also considered, based on SEACTN data the absolute risk of a severe outcome, defined as death or hospitalisation for longer than 3 days or persistent symptoms at 28 days post presentation, is minimal (approximately 1.5%).

### Description of statistical analyses

The primary endpoint is the proportion of patient consultations for AFI which result in antibiotic prescription. The primary comparison between the treatment arms will be a logistic regression with the treatment assignment as a fixed effect, and the PHC as a random effect. P values (two-sided) below 0.05 are considered significant. A two-sided 95% CI for the OR of being prescribed antibiotics will be calculated.

The primary endpoint (proportion of patients given an antibiotic prescription) will also be investigated by subgroup to assess whether any change in antibiotic prescribing is homogeneous across subgroups. Specifically, intervention effects and appropriate tests for heterogeneity will be calculated in the following predefined subgroups:

- Age: younger children aged ≥1 to <5 years, older children aged 5 to <15 years and adults aged ≥15 years.
- Sex at birth: male or female.
- Patients with a documented fever or hypothermia at presentation versus patients without.

The secondary endpoints will be analysed in a similar manner to the primary endpoint. A detailed Statistical Analysis Plan will be developed prior to the completion of recruitment for the study.

### Data management

Data will be captured electronically through the EDAM app and submitted to a central server. A data dictionary will be constructed in which all variables are clearly explained. A logbook of patients screened and recruited will also be maintained.

Patients will be assigned a unique composite ID number each time they are enrolled. They will be identified only by their unique ID number on study documents, in data entry and in any electronic databases in order to protect their privacy. The only patient-identifying data entered in the logbooks will be the names of patients, their villages of residence and their telephone numbers, for the purposes of carrying out follow-up. No other identifiers will be collected, and these details will not be entered into any databases held or accessed by the study team. All documents will be stored securely and only accessible by study staff and authorised personnel. Any scientific publications or reports will not identify any individual participant/s by name or initials.

Electronic data will not be modifiable by the healthcare worker after submission, although authorised users, for example, site research staff will be able to do so for data cleaning purposes. Access to the data will be password protected. Direct access will be granted to authorised representatives from the University of Oxford, the study site partners and the respective national ethics committees for monitoring and/or audit of the study to ensure compliance with regulations.

The database and all electronic data will be routinely backed up. In accordance with MORU Standard Operating Procedures, deidentified electronic data will be stored indefinitely on the central server, while paper records will be preserved for 5 years.

Healthcare workers will be trained prior to study commencement on collection of data using the version of the EDAM app relevant to their PHC. Training materials, for example, videos will be uploaded to the tablet devices in case they need to refer to these.

### Data quality assurance

Data validation will be performed to identify errors and thus ensure completeness, validity and data accuracy.

Electronic data checks and field restrictions will prevent errors with extreme values being entered, for instance by having prespecified ranges for vital sign measurements. Further internal checks of the entered data will be done to look for outliers and errors. Additionally, because data will be transmitted in real time or near-real time to the central server, data cleaning will occur prior to the conclusion of the study allowing for earlier detection and correction of errors if necessary.

The study will be conducted in compliance with this protocol, International Conference on Harmonization Guidelines for GCP and any applicable regulatory requirement(s). Staff involved in data collection, interviews, transcription and data coding will be appropriately qualified to perform assigned tasks and will be trained on study procedures prior to start of the study. Monitoring will be overseen by the MORU Clinical Trials Support Group to ensure compliance to the study protocol and applicable guidelines and regulations.

### Safety considerations

The risks of participation are low. First, measurement of oxygen saturation using pulse oximetry is now considered the ‘fifth vital sign’ and is recommended by the WHO when possible in rural LMIC primary care settings,^34^,^36^ therefore its use is not controversial. Second, symptom-based and vital sign-based diagnostic algorithms, such as the Integrated Management of Childhood Illness guideline, are also widely used in clinical practice. Lastly, MORU has previously conducted a large clinical trial on CRP-guided treatment in patients with AFI with extensive patient follow-up, demonstrating that it is effective in reducing antibiotic prescribing and did not adversely affect patient outcomes.^37^ A decision support tool integrating all three would, therefore, not be expected to pose any elevated risk to patient safety. Healthcare workers in the intervention arm are also able to use their discretion if they do not agree with the management recommended by EDAM. Additionally, as an extra safeguard, they will be trained to provide safety netting to all patients they recruit.

Furthermore, in line with the principles guiding data and safety monitoring in pragmatic clinical trials,^38^ an independent Data and Safety Monitoring Board (DSMB) was assembled prior to the commencement of the trial. The DSMB has reviewed the protocol and has access to summary data in real time through the electronic trial dashboard; given the anticipated short duration of this study, no interim analyses will be provided, although it may request additional information if necessary. There are three independent and voting members of this committee, with the chair being medically qualified and one of the other members being a statistician. The DSMB will determine meeting frequency at its first meeting.

Given the pragmatic nature of this simple intervention, remote and on-site monitoring will be undertaken in accordance with a monitoring plan developed prior to study commencement to confirm the quality of the research performed to assess and ensure the robustness of the trial results.

### Status and timeline

This study commenced in late May 2024 and will run until the recruitment target for each cluster is met, which is likely to be approximately 6–9 months.

## Ethics and dissemination

###  Ethical approval

The study sponsor is the University of Oxford, and the protocol has been approved by the Oxford University Tropical Research Ethics Committee (OxTREC, reference 550-23). The protocol has also been approved by the Cambodian National Ethics Committee for Health Research (NECHR, reference 395-NECHR). Permission to carry out the study has been obtained from the local health authority.

Any amendments and modifications will be submitted to the above-mentioned boards for review and approval. No changes in protocol conduct will be implemented until approvals of amendments by all relevant boards are obtained.

### Trial registration

This study has been registered in the International Standard Randomized Controlled Trial Number (ISRCTN) Registry (reference number ISRCTN15157105).^39^

### Dissemination

The results of the study will be published in appropriate peer-reviewed journals. Authorship eligibility will be based on the criteria set by the International Committee of Medical Journal Editors,^40^ with author contributions described using the Contributor Roles Taxonomy.^41^ There are no plans to use professional medical writers in the drafting of the manuscript containing the results of the study.

## supplementary material

10.1136/bmjopen-2024-089616 online supplemental file 1

10.1136/bmjopen-2024-089616 online supplemental file 2
