# Supplementary material for: Evaluation of an electronic clinical decision support algorithm to improve primary care management of acute febrile illness in rural Cambodia: protocol for a cluster-randomised trial
Source: BMJ Open. 2024 Oct 18;14(10):e089616. doi: 10.1136/bmjopen-2024-089616 (PMC11492946; doi:10.1136/bmjopen-2024-089616)
Supplement: online supplemental file 1 [file bmjopen-14-10-s001.pdf]

PRECIS-2 toolkit

We would be very grateful if users would give us feedback on using PRECIS-2: just click on "Contact us". These PRECIS-2 criteria are constantly being reviewed and we welcome your input.

How to use PRECIS-2 - Designing trials that are fit for purpose

We think there are four steps to using PRECIS-2, which may be iterative depending on what you discover after going through the steps.

Step 1: Why are you doing your trial?

Your first step is to be clear about why you are doing your trial. Are you:

1. Aiming to take an explanatory approach to answer the question 'Can this intervention work under ideal conditions?'
2. Aiming to take a pragmatic approach and answer the question 'Does this intervention work under usual conditions?'

Both approaches to trial design have their place but trialists should be clear which path they are on. As Schwartz and Lellouch pointed out, trialists have often taken the first approach by default rather than as a considered judgement.

Step 2: Consider your trial design choices for each of the nine PRECIS-2 domains

This step is explained in more detail for each domain later on.

Step 3: Score 1 to 5 for these choices made in Step 2 and/or mark on the PRECIS-2 wheel

Having considered your design choices in Step 2, the PRECIS-2 wheel is used to record how pragmatic or explanatory these choices are for each domain. Each domain is a 5-point Likert scale:

1. Very explanatory
2. Rather explanatory
3. Equally pragmatic/explanatory
4. Rather pragmatic
5. Very pragmatic

A table can be used in conjunction with the PRECIS "wheel" or instead of the wheel to give rationale for scores. You can use this to assist discussion with trial collaborators.

Step 4: Review your PRECIS-2 wheel

Review your design choices (Step 2) on the PRECIS-2 wheel to see whether they will produce a trial that will support the aim identified in Step 1. Go back to Step 2 and modify your design choices if required.

PRECIS-2 wheel

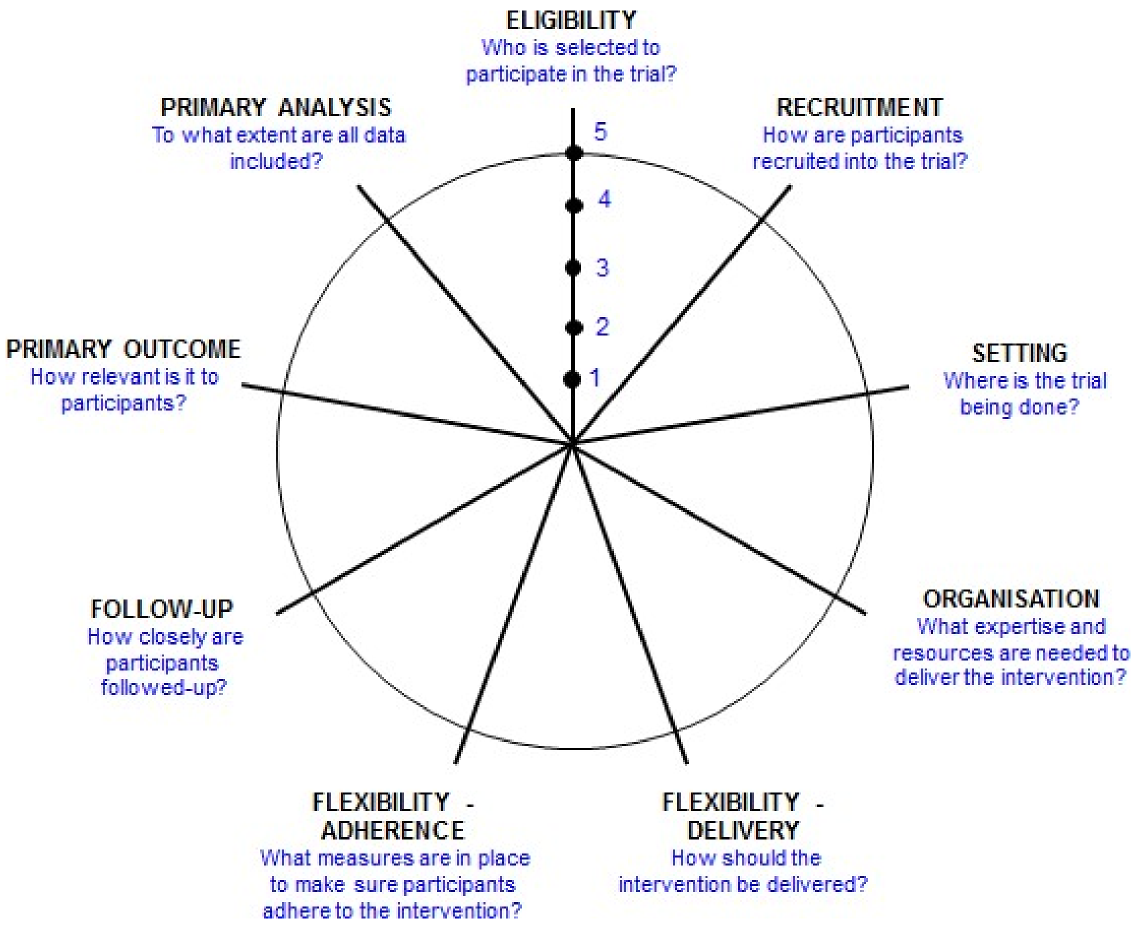

Table 2: PRECIS-2 scores for trial domains

	Domain	Score	Rationale
1	Eligibility Criteria	5	The goal of the intervention is for it to be incorporated as part of routine care for patients with acute febrile illness in rural South and Southeast Asian primary health centres, therefore all patients who present with acute fever will be eligible. The only exception is neonates, for whom fever necessitates referral to secondary care as per well-established guidance.
2	Recruitment Path	4	Patients will be pragmatically recruited as they present to study facilities, with written consent waivers sought from the relevant ethics committees. Healthcare workers will, however, receive compensation for the time taken to enroll and follow up patients, at sites where this is necessary to the successful running of the trial. This may involve per capita payment for each recruited patient.
3	Setting	5	The trial and usual care settings are identical i.e., rural South and Southeast Asian primary health centres.
4	Organisation intervention	5	The trial uses identical organization to usual care, in that the same healthcare workers who normally work at the primary health centres involved in the trial will be those using the intervention (or continuing with the current standard of care if working at a primary health centre in the control arm).
5	Flex of experimental intervention – Delivery	5	While healthcare workers in the intervention arm are recommended to follow the recommendations of the EDAM app, there is no compulsion to do so and their clinical judgement will remain paramount.
6	Flex of experimental intervention – Adherence	5	While healthcare workers in the intervention arm are recommended to follow the recommendations of the EDAM app, there is no compulsion to do so and their clinical judgement will remain paramount.
7	Follow up	3	Not all patients would be routinely followed up, but because some of the secondary outcomes involve events which occur at later time points e.g., recovery at day 7, telephone follow-up is necessary. This follow-up method was chosen to ensure that the nature of follow up is well-balanced between pragmatic and explanatory.
8	Outcome	5	The primary outcome is the proportion of patients prescribed antibiotics. This is of importance to the participants (patients), as well as healthcare workers and the wider health system.
9	Analysis	5	An intention-to-treat approach will be taken for the analysis of the primary outcome, although a per protocol analysis will also be performed in parallel.

The PRECIS-2 Domains

The NINE *PRECIS-2* domains are:

- ***Eligibility*** –to what extent are the participants in the trial similar to those who would receive this intervention if it was part of usual care? For example, score 5 for very pragmatic criteria essentially identical to those in usual care; score 1 for a very explanatory approach with lots of exclusions (e.g. those who don't comply, respond to treatment, or are not at high risk for primary outcome, are children or elderly), or uses many selection tests not used in usual care.
- ***Recruitment*** - how much extra effort is made to recruit participants over and above what that would be used in the usual care setting to engage with patients? For example, score 5 for very pragmatic recruitment through usual appointments or clinic; score 1 for a very explanatory approach with targeted invitation letters, advertising in newspapers, radio plus incentives and other routes that would not be used in usual care.
- ***Setting*** – how different is the setting of the trial and the usual care setting? For example, score 5 for a very pragmatic choice using identical settings to usual care; score 1, for a very explanatory approach with only a single centre, or only specialised trial or academic centres.
- ***Organisation*** – how different are the resources, provider expertise and the organisation of care delivery in the intervention arm of the trial and those available in usual care? For example, score 5 for a very pragmatic choice that uses identical organisation to usual care; score 1 for a very explanatory approach if the trial increases staff levels, gives additional training, require more than usual experience or certification and increase resources.
- ***Flexibility (delivery)*** – how different is the flexibility in how the intervention is delivered and the flexibility likely in usual care? For example, score 5 for a very pragmatic choice with identical flexibility to usual care; score 1 for a very explanatory approach if there is a strict protocol, monitoring and measures to improve compliance, with specific advice on allowed co-interventions and complications.
- ***Flexibility (adherence)*** - how different is the flexibility in how participants must adhere to the intervention and the flexibility likely in usual care? For example, score 5 for a very pragmatic choice involving no more than usual encouragement to adhere to the intervention; score 1 for a very explanatory approach that involves exclusion based on adherence, and measures to improve adherence if found wanting. In some trials eg surgical trials where patients are being operated on or Intensive Care Unit trials where patients are being given IV drug therapy, this domain is not applicable as there is no compliance issue after consent has been given, so this score should be left blank.
- ***Follow-up*** - how different is the intensity of measurement and follow-up of participants in the trial and the likely follow-up in usual care? For example, score 5 for a very pragmatic approach with no more than usual follow up; score 1 for a very explanatory approach with more frequent, longer visits, unscheduled visits triggered by primary outcome event or intervening event, and more extensive data collection.
- ***Primary outcome*** – to what extent is the trial's primary outcome relevant to participants? For example, score 5 for a very pragmatic choice where the outcome is of obvious importance to participants; score 1 for a very explanatory approach using a surrogate, physiological outcome, central adjudication or use assessment expertise that is not available in usual care, or the outcome is measured at an earlier time than in usual care.
- ***Primary analysis*** – to what extent are all data included in the analysis of the primary outcome? For example, score 5 for a very pragmatic approach using intention to treat with all available data; score 1 for a very explanatory analysis that excludes ineligible post-randomisation participants, includes only completers or those following the treatment protocol.

Notes

“Participants" include patients or other individual recipients of an intervention, and/or providers of the intervention. This may include individual participants and/or one or more levels of clusters. For example, in a trial of a continuing education intervention, participants may be health professionals and trained instructors and the trial may be randomised into clusters at the level of the instructor.

During the design process, if there is uncertainty over how explanatory or pragmatic a domain is, then we suggest the score for this domain should be left blank. This will then highlight uncertainty and encourage discussion. If PRECIS-2 is used to look at how pragmatic included trials are in systematic reviews then a score of 3 may be chosen if there is inadequate information. This is different to the “3 = equally pragmatic/explanatory”.

Example: PRECIS-2 wheel.

Little P, Moore M, Kelly J, Williamson I, Leydon G, McDermott L, Mullee M, Stuart B: Ibuprofen, paracetamol, and steam for patients with respiratory tract infections in primary care: pragmatic randomised factorial trial. *BMJ* 2013, 347:f6041.

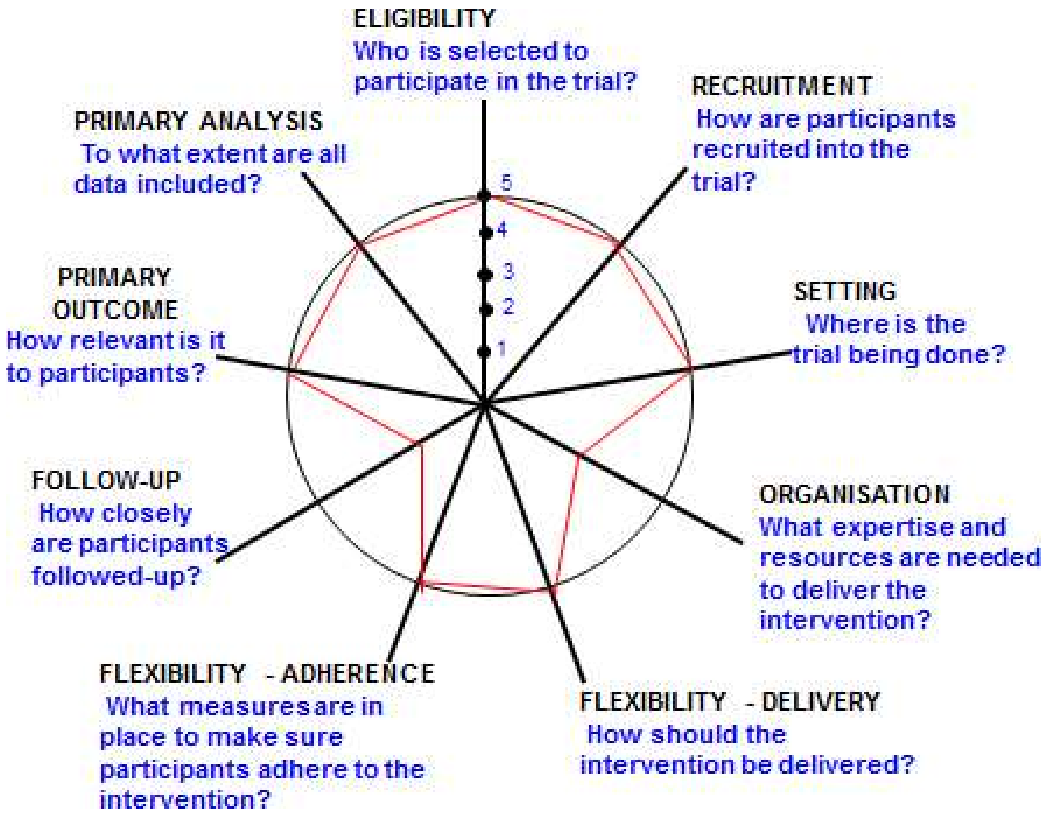

Table – PRECIS-2 scores for trial domains

	Domain	Score	Rationale
1	Eligibility Criteria	5	Patients ≥ 3 with acute respiratory tract infections. Exclusions not common cold but severe respiratory tract infections, peptic ulcer and asthma. But even asthma patients who were not sensitive to ibuprofen or aspirin eligible. Pregnant or breast feeding women are discouraged from taking ibuprofen when pregnant but can take as low a dose as possible of Paracetemol but as both being tested in the trial cannot include this group as participants. If include visually impaired increase organisation of trial (as in carers have to assist diary completion) but that would be norm and usual if prescribed ibuprofen or Paracetemol or steam treatment in usual care, so just easier for trialists to exclude. Small proportion of participants – so why exclude? Depressed and people with psychosis unreliable diary fillers, concerned with other things.
2	Recruitment Path	5	Patients presenting to GP or nurse in primary care with respiratory tract infections as diagnosed by health professional. Easy to recruit to but time pressures in busy winter clinics prevented recruitment and meant non-recruitments logs poorly completed.
3	Setting	5	Identical setting to usual care setting - primary care where patients usually go for advice and treatment.
4	Organisation intervention	3	Information sheets given out to participants. This is not usual practice, would it be if trial had shown this helped? Need more staff for follow up of trial participants to get diary results so more extensive data collection than usual. Otherwise no difference in provider expertise and organisation of care delivery.
5	Flex of experimental intervention – Delivery	5	Full flexibility

Table – PRECIS-2 scores for trial domains (continued)

6	Flex of experimental intervention – Adherence	5	Compliance measured with diary – "reasonably good" participants free to "not-comply" with advice. Little impact of advising patients to take analgesics regularly. No measures to improve compliance
7	Follow up	2	Participants Followed up one month – usual care no follow up. Patients come back to GP if problems.<br><br>Trial more extensive data colleaction - patients telephoned day two to three to check for any problems with diary completion. If no diary after 2 weeks then mailed reminder and then phoned as necessary to document outcomes using validated questionnaire.
8	Outcome	5	Very relevant to participants - Mean symptom severity on days 2-4. Symptoms 0 (no problem) to 6 (as bad as they can be 7 in abstract). Completed in diary at end of day – children with parents or participants.
9	Analysis	5	ITT no matter whether compliance.
