# Supplementary material for: Evaluation of an electronic clinical decision support algorithm to improve primary care management of acute febrile illness in rural Cambodia: protocol for a cluster-randomised trial
Source: BMJ Open. 2024 Oct 18;14(10):e089616. doi: 10.1136/bmjopen-2024-089616 (PMC11492946; doi:10.1136/bmjopen-2024-089616)
Supplement: Supplementary file 3 [file bmjopen-2024-089616.reviewer_comments.pdf]

PEER REVIEW HISTORY

BMJ Open publishes all reviews undertaken for accepted manuscripts. Reviewers are asked to complete a checklist review form and are provided with free text boxes to elaborate on their assessment. These free text comments are reproduced below.

ARTICLE DETAILS

Title (Provisional)

Evaluation of an electronic clinical decision support algorithm to improve primary care management of acute febrile illness in rural Cambodia: Protocol for a cluster-randomized trial

Authors

Chew, Rusheng; Wynberg, Elke; Liverani, Marco; Rekol, Huy; Nguon, Chea; Dysoley, Lek; Vanna, Moul; Callery, James John; Mishra, Abhijit; Adhikari, Bipin; Tripura, Rupam; Chandna, Arjun; Fegan, Greg; Waithira, Naomi; Maude, Richard James; Day, Nicholas P.J.; Peto, Thomas Julian; Lubell, Yoel

---

VERSION 1 - REVIEW

**Reviewer**	**1**
**Name**	**Attai, Kingsley**
**Affiliation**	**Ritman University**
**Date**	**19-Jun-2024**
**COI**	**Yes**

1.The authors should elaborate on the challenge of electronic clinical decision support algorithm to improve primary care management

2.The authors should better investigate the existing literature review and update this Section.(https://doi.org/10.1177/14604582241260659; https://doi.org/10.3390/tropicalmed8070352; https://doi.org/10.1016/j.ceh.2024.05.001)

3. The authors did not elucidate how their research builds upon previous work in electronic clinical decision support algorithm to improve primary care management

4. The quality of Figures should be improved

**Reviewer**	**2**
**Name**	**Niessen, Louis**
**Affiliation**	**Liverpool School of Tropical Medicine, Health Economics**
**Date**	**20-Jun-2024**

COI **None.**

The protocol paper describes a relatively straightforward pragmatic trial on acute fever among young children in rural Cambodia, with primary outcome reduction in antibiotics prescribing. It should be published as this is an important matter at individual and societal level, addressing potential pitfalls, is well-written, and addresses bias in the consent procedure.

Points to address in minor revisions:

1) South and South-East Asia in the title is very ambitious. It could be maintained if there would be a (base-line) comparison at least of specific disease burden of the SEACTN countries. I assume this varies a lot.

2) There still is a big Hawthorne effect possible, as is known about addressing antibiotics prescribing. Describe was is done or not done in the control arm (min intervention, visibility of research effort etc).

3) How are the selected PHCs chosen? Any randomness like from a list of elegible centres? Convenience sample? Potential bias? Matches with control arm?

4) Are contextual factors studied and taken into account? Malaria programme activities? Private sector consultations or direct pharmacy visits? Availability of market medication?

5) How involved has the local population been in formulation of the study, study design , study implementation? A presentation to (local) health workers is not enough in these days of (9nternal) decolonisation.

6) Enitan Carrol, to my knowledge, doesn't work at LSTM but at the University of Liverpool.

Good luck with the study!

**Reviewer**	**3**
**Name**	**Asuquo, Daniel E.**
**Affiliation**	**University of Uyo, Department of Information Systems**
**Date**	**04-Jul-2024**
**COI**	**There is no competing interest**

The authors can cite additional literature on febrile disease diagnosis in LMICs beyond malaria and the use of Rapid Diagnostic Test Kits e.g.

(i) Asuquo, D., Attai, K., Obot, O., Ekpenyong, M., Akwaowo, C., Arnold, K. and Uzoka, F-M. (2024). Febrile Disease Modeling and Diagnosis System for Optimizing Medical Decisions in Resource-scarce Settings, Clinical eHealth, 7, 52-76, https://doi.org/https://doi.org/10.1016/j.ceh.2024.05.001

(ii) Asuquo, D., Attai, K., Johnson, E., Obot, O., Adeoye, O., Akwaowo, C., Ekpenyong, N., Isiguzo, C., Ekanem, U., Motilewa, O., Dan, E., Umoh, E., Ekpin, V., Uzoka, F-M. (2024). Multi-criteria Decision Analysis Method for Differential Diagnosis of Tropical Febrile Diseases, Health Informatics Journal, 30(2), 1-41, https://doi.org/10.1177/14604582241260659

VERSION 1 - AUTHOR RESPONSE

Reviewer 1 comments

- *The authors should elaborate on the challenge of electronic clinical decision support algorithm to improve primary care management.*

  Thank you for this suggestion. We have added the following sentences that describe the barriers to the development and adoption of electronic clinical decision support algorithms (eCDSAs) to AFI management in the rural Southeast Asian context, most of which are likely universal (lines 170–175):

  "This may be due to barriers noted in evaluations of eCDSAs elsewhere, such as lack of technical skills among healthcare workers, heavy workloads, perceived threats to healthcare worker autonomy, and inability of the eCDSA to be integrated into clinical workflows effectively.[9 10] Lack of patient trust in eCDSA-generated recommendations and concerns regarding data privacy and security may also be additional inhibiting factors.[11]"

- *The authors should better investigate the existing literature review and update this Section. (https://doi.org/10.1177/14604582241260659; https://doi.org/10.3390/tropicalmed8070352; https://doi.org/10.1016/j.ceh.2024.05.001)*

  Thank you, we have added these references (refs. 17–19) as suggested, noting that these are potential improvements to the current version of the EDAM app (lines 211–213):

  "The results will provide a solid foundation for future iterations of the app, which may include more sophisticated technologies such as machine learning as has been done for eCDSAs for AFI in Nigeria.[17-19]"

- *The authors did not elucidate how their research builds upon previous work in electronic clinical decision support algorithm to improve primary care management*

  Thank you for this suggestion to highlight the novelty and importance of our work. We have described how the EDAM app improves upon previous eCDSAs for AFI (lines 201–205):

  "Besides being the first eCDSA for AFI in the rural Southeast Asian primary care context, and by incorporating point-of-care diagnostic tools other than malaria rapid tests and having antimicrobial stewardship as a guiding principle in its development, EDAM is an improvement on current guideline-based eCDSAs for AFI and is in line with the target

product profile for such eCDSAs developed by the Foundation for Innovative New Diagnostics (FIND).[16]"

- *The quality of Figures should be improved*

  The figures have been re-created with higher resolutions, which should result in better clarity (although we believe that this will be corrected if necessary as part of the article production process).

Reviewer 2 comments

- *The protocol paper describes a relatively straightforward pragmatic trial on acute fever among young children in rural Cambodia, with primary outcome reduction in antibiotics prescribing. It should be published as this is an important matter at individual and societal level, addressing potential pitfalls, is well-written, and addresses bias in the consent procedure.*

  Thank you for this very encouraging comment, and for the subsequent recommendations for minor edits to the manuscript.

- *South and South-East Asia in the title is very ambitious. It could be maintained if there would be a (base-line) comparison at least of specific disease burden of the SEACTN countries. I assume this varies a lot.*

  Many thanks for pointing this out, we agree. Since the trial is only being run in Cambodia, we have changed 'South and Southeast Asia' to 'Cambodia'.

- *There still is a big Hawthorne effect possible, as is known about addressing antibiotics prescribing. Describe was is done or not done in the control arm (min intervention, visibility of research effort etc).*

  Thank you for reminding us of this issue, with which we fully agree. We have endeavoured to maintain clinical practice and workflows at control arm PHCs as close to the current standard of care as possible. The only extra activity required of healthcare workers in the control arm is screening and data collection, although there are also posters displayed in the health centres explaining the trial. This has been explained in the Methods (lines 405–412 and 547–548):

  "To maintain clinical practice and workflows at control arm PHCs as close to the current standard of care as possible, the only extra activity for the trial required of healthcare workers in the control arm is screening and data collection. They will receive a simplified version of the EDAM app which will allow them to log patient demographic details as well as conduct patient screening and enrolment, but does not contain the clinical decision support algorithms present in the version used in the intervention arm. Control arm healthcare workers will receive training in the use of the simplified EDAM app, but not in the clinical procedures and use of point-of-care aids that healthcare workers in the intervention arm are taught."

  "Information on EDAM for patients will be displayed in all study PHCs in the form of posters…"

- *How are the selected PHCs chosen? Any randomness like from a list of elegible centres? Convenience sample? Potential bias? Matches with control arm?*

  We have stated the eligibility criteria for primary health centres to be considered for inclusion in the trial (lines 278–283); however, in response to this comment we have also now detailed the method by which PHCs are randomised (lines 564–568 and 577–582):

  "PHCs participating in SEACTN will also participate in this study if they meet criteria for inclusion. Other PHCs will be selected for inclusion in this study, if required, to make up a total of 15 PHCs in each of the control and intervention arms per country, on the assumption that their antibiotic prescription rates are also broadly similar. The PHCs will be drawn from across three Operational Districts as defined by the Battambang Provincial Health Department, with ten selected from each Operational District."

  "Stratified cluster randomisation of PHCs by Operational District was performed; as mentioned previously, ten clusters were included per district. A pseudo-randomisation list of allocations to one of two possible arms ('A' or 'B') was generated using the ralloc package in Stata 18.0 (StataCorp, Texas, USA). This list was merged 1:1 with a list of PHCs. Assignment of arms 'A' and 'B' as either intervention or control was performed using the flip of a coin, with arm 'A' designated as the intervention arm if the coin flip came up heads and vice versa."

- *Are contextual factors studied and taken into account? Malaria programme activities? Private sector consultations or direct pharmacy visits? Availability of market medication?*

  We agree that contextual factors are very important and, to the best of our ability, these have been considered in the planning and implementation of the trial. For example, as already stated there is continued emphasis on testing all acutely febrile patients recruited for malaria as per the recommendation of the Cambodian National Malaria Control Program. We have clarified this decision further to emphasise the rationale (lines 360–363):

  "Eligible patients will have a malaria rapid test administered early following measurement of vital signs, as is routine for all febrile patients at the study sites. This is based on the recommendation of the Cambodian National Malaria Control Program, since a continued comprehensive testing strategy is crucial to eradication efforts."

  While we do not study drivers behind decisions to prescribe antibiotics or purchase them privately, we do collect these data as part of the outcome measures (lines 505–507):

  "5. The proportion of patients prescribed antibiotics by a healthcare provider or who independently purchased antibiotics during the follow-up period, as determined by self-report, in intervention and control PHCs."

  Importantly, interpretation of the trial results will make reference to a separate work undertaken to understand the primary healthcare context in SEACTN countries, including Cambodia (lines 515–519):

  "Interpretation of the outcomes will consider the contextual factors inherent to the Cambodian primary care landscape, as described by Chew et al.[31] In addition, the usability and acceptability of the app will be measured using structured interviews conducted with

the healthcare workers in the intervention arm; this study does not form part of this protocol and will have its own ethical approval."

- *How involved has the local population been in formulation of the study, study design, study implementation? A presentation to (local) health workers is not enough in these days of (9nternal) decolonisation.*

  We agree that the decolonisation of global health is a priority. Nevertheless, given that the trial focuses on clinical management, most of the public engagement work has been with local medical staff who devised the contextually-appropriate clinical management plans as well as senior physicians from the Battambang Provincial Health Department, who have endorsed them and the study design. We have also obtained the endorsement of the Battambang provincial government for the trial. These points have been made clearer in the manuscript (lines 528–530):

  "These management plans, as well as the overall design of the study, were further reviewed and endorsed by senior medical staff of the Battambang Provincial Health Department, and support for the trial was also obtained from the provincial government."

  As described in the manuscript, the implementation of the trial is by local primary health centre workers with the assistance of the local non-governmental organisation Action for Health Development (AHEAD) and the Provincial Health Department. The following sentence has been added to highlight local involvement even further:

  "Day-to-day field activities and monitoring will be undertaken by staff of MORU, AHEAD, and the Provincial Health Department." (lines 542–543)

- *Enitan Carrol, to my knowledge, doesn't work at LSTM but at the University of Liverpool.*

  Thank you for pointing this out, we have made the necessary correction (line 187).

Reviewer 3 comments

- *Results: The authors can cite additional literature on febrile disease diagnosis in LMICs beyond malaria and the use of Rapid Diagnostic Test Kits e.g.*
- *(i) Asuquo, D., Attai, K., Obot, O., Ekpenyong, M., Akwaowo, C., Arnold, K. and Uzoka, F-M. (2024). Febrile Disease Modeling and Diagnosis System for Optimizing Medical Decisions in Resource-scarce Settings, Clinical eHealth, 7, 52-76, https://doi.org/https://doi.org/10.1016/j.ceh.2024.05.001*
- *(ii) Asuquo, D., Attai, K., Johnson, E., Obot, O., Adeoye, O., Akwaowo, C., Ekpenyong, N., Isiguzo, C., Ekanem, U., Motilewa, O., Dan, E., Umoh, E., Ekpin, V., Uzoka, F-M. (2024). Multi-criteria Decision Analysis Method for Differential Diagnosis of Tropical Febrile Diseases, Health Informatics Journal, 30(2), 1-41, https://doi.org/10.1177/14604582241260659*

  Thank you, these references have been added (we note that Reviewer 1 has also recommended these).

VERSION 2 - REVIEW

**Reviewer**	**2**
**Name**	**Niessen, Louis**
**Affiliation**	**Liverpool School of Tropical Medicine, Health Economics**
**Date**	**26-Sep-2024**
**COI**	

The authors have responded to my review points well and adapted the manuscript accordingly. It can be published.

I am somewhat disappointed by the relatively low level of community and patients / families involvement. Just giving this as a task to the involved health workers is not enough. It should be structured within the trial organization and be less top-down (or old-style colonial).

VERSION 2 - AUTHOR RESPONSE

Reviewer 2 comment

- *The authors have responded to my review points well and adapted the manuscript accordingly. It can be published.*
  I am somewhat disappointed by the relatively low level of community and patients / families involvement. Just giving this as a task to the involved health workers is not enough. It should be structured within the trial organization and be less top-down (or old-style colonial).

  Thank you. For the record, this study builds on several years of collaboration between the Cambodian non-governmental organisation Action for Health Development (AHEAD) and the Battambang Provincial Health Department. The planning and implementation of the trial involved community health workers, AHEAD, Cambodian medical specialists, the Battambang provincial and also national authorities. These included workshops that informed the development of the intervention – this process will be written up separately as it will be informative and, we hope, an example of good community engagement for others wishing to conduct similar research. Of course, we could always have done more, but we did far more than is done in most studies where community engagement is concerned.
